# Generation of Transfer-DNA-Free Base-Edited Citrus Plants

**DOI:** 10.3389/fpls.2022.835282

**Published:** 2022-03-15

**Authors:** Berta Alquézar, Stefania Bennici, Lourdes Carmona, Alessandra Gentile, Leandro Peña

**Affiliations:** ^1^Laboratório de Biotecnologia Vegetal, Pesquisa, and Desenvolvimento, Fundo de Defesa da Citricultura (Fundecitrus), Araraquara, Brazil; ^2^Instituto de Biología Molecular y Celular de Plantas, Consejo Superior de Investigaciones Científicas, Universidad Politécnica de Valencia, Valencia, Spain; ^3^Department of Agriculture, Food, and Environment, University of Catania, Catania, Italy

**Keywords:** citrus, CRISPR, T-DNA free, APOBEC1, biotechnology, ALS

## Abstract

To recover transgenic citrus plants in the most efficient manner, the use of selection marker genes is essential. In this work, it was shown that the mutated forms of the acetolactate synthase (*ALS*) gene in combination with the herbicide selection agent imazapyr (IMZ) added to the selection medium may be used to achieve this goal. This approach enables the development of cisgenic regenerants, namely, plants without the incorporation of those bacterial genes currently employed for transgenic selection, and additionally it allows the generation of edited, non-transgenic plants with altered endogenous *ALS* genes leading to IMZ resistance. In this work, the citrus mutants, in which *ALS* has been converted into IMZ-resistant forms using a base editor system, were recovered after cocultivation of the explants with *Agrobacterium tumefaciens* carrying a cytidine deaminase fused to nSpCas9 in the T-DNA and selecting regenerants in the culture medium supplemented with IMZ. Analysis of transgene-free plants indicated that the transient expression of the T-DNA genes was sufficient to induce *ALS* mutations and thus generate IMZ-resistant shoots at 11.7% frequency. To our knowledge, this is the first report of T-DNA-free edited citrus plants. Although further optimization is required to increase edition efficiency, this methodology will allow generating new citrus varieties with improved organoleptic/agronomic features without the need to use foreign genes.

## Introduction

Citrus are susceptible to biotic and abiotic stresses, which can compromise their yield, quality, and survival in the case of being affected by aggressive diseases. Developing new varieties resistant to adverse environmental conditions, pests, and pathogens by conventional breeding is hindered by citrus long juvenility and complex reproductive characteristics (sterility, high heterozygosity, sexual incompatibility, parthenocarpy, and facultative apomixis). Although citrus genetic transformation of some citrus varieties is feasible, there are few transgenic cultivars resistant/tolerant to important citrus diseases. For example, resistance to the oomycete *Phytophthora* spp. (Fagoaga et al., 2001; Sandhu et al., 2019) and the fungus *Penicillium digitatum* (Rodríguez et al., 2014) has been achieved successfully. Transgenic genotypes resistant to tristeza and psorosis causal viruses have also been developed (Soler et al., 2012; De Francesco et al., 2020), as well as to the bacterium responsible for citrus canker, *Xanthomonas citri* spp. Citri (Hao et al., 2016; Kobayashi et al., 2017). However, field trials of these resistant cultivars are scarce, and no commercial transgenic plantings have been established thus far. This may be related to regulatory burdens and consumers’ concerns about genetic modified organisms (GMOs). The most devastating disease currently threatening citrus industries worldwide is huanglongbing (HLB). HLB has not yet been cured, and attempts to minimize its damages have huge environmental and economic costs (Bassanezi et al., 2020). It has been proposed that genetically modified HLB-resistant cultivars are the main feasible option to obtain durable and sustainable control of this disease (Yan et al., 2015; McCollum and Baldwin, 2016; NASC, 2018; Cochrane and Shade, 2019; Wang, 2020; Alquezar et al., 2021).

The recently developed genome editing technologies open up opportunities to engineer citrus types without the introduction of foreign DNA in their genomes, likely overcoming consumer hesitance and legal restrictions associated with transgenic crops. Clustered regularly interspaced short palindromic repeats (CRISPR) and CRISPR-associated (Cas) nucleases have already been established in many plants. In herbaceous crops, it has been used to generate transgenic plants harboring CRISPR/Cas components required to edit the genome, and after targeted edition has taken place, T-DNA is bred out to obtain edited non-transgenic lines by sexual crossings. This strategy has already been used to generate rice, wheat, and maize edited lines with improved yield, quality, or disease resistance. Currently, hundreds of crop edited varieties are considered non-regulated by the US Department of Agriculture, showing improved agronomic performance and availability for commercial cultivation in the United States of America (USA) (Zhu et al., 2020). By means of genetic modification, the ability of Cas9 from different sources has already been shown to edit the citrus genome. *Streptococcus pyogenes* Cas9 (SpCas9) has been used to disrupt the phytoene desaturase (*PDS*) gene in Carrizo citrange to obtain albino chimeric plants (LeBlanc et al., 2018), and the *LATERAL ORGAN BOUNDARIES 1* (*LOB1*) promoter or its coding sequence has been mutated in sweet orange and grapefruit to generate resistance to citrus canker (Jia et al., 2017b; Peng et al., 2017; Jia and Wang, 2020). *Lachnospiraceae bacterium* Cas12a (LbCas12a) was also used to edit the genomes of Carrizo citrange and grapefruit transgenic lines (Jia et al., 2019). As in other plants, the edition efficiency is variable between citrus transgenic lines, ranging from 11% to almost 90%. However, citrus heterozygosity and complex reproductive characteristics precluded the acquisition of foreign DNA-free edited cultivars. Even if the variety to edit was one of the few self-pollinated, monoembryonic varieties of commercial interest, the elimination of T-DNA through sexual crosses would entail an extremely lengthy time due to the long juvenile period of this crop. More importantly, the siblings would be hybrids, likely lacking the elite attributes of the original parent genotypes. Thus, T-DNA-free methodologies are required to develop non-transgenic edited citrus cultivars. Although these techniques present advantages such as reduced off-target mutations and fewer regulatory concerns, they are impeded by their low efficiency (Zhu et al., 2020). For example, in citrus, *PDS*-SpCas9 and *LOB1*-LbCas12a edition efficiencies dropped to 3.2–3.9 and 2%, respectively, when provided via *Xanthomonas citri* (Xcc)-facilitated agroinfiltration to enhance transient expression (Jia and Wang, 2014; Jia et al., 2019). Different strategies are being investigated in citrus to increase the efficiency of the different Cas9 enzymes already available to address foreign DNA-free edition techniques. For example, it has been reported that SpCas9 edition efficiency is improved at 37°C (LeBlanc et al., 2018), and more recently, the use of citrus U6 promoters and tRNA-mediated edition has allowed to greatly improve biallelic and homozygous mutation rates up to 44.4 and 11.1%, respectively (Huang et al., 2020). In addition, successful non-transgenic edition of embryogenic protoplasts has already been accomplished in citrus, but no plants were regenerated from them (Huang et al., 2020). However, all progress relies on the use of exogenous selection markers [nopaline synthase-neomycin phosphotransferase II gene (*nptII*), β-glucuronidase gene (*GUS*), and green fluorescent protein coding gene (*GFP*)] during *in vitro* culture stages to select and regenerate plants from transgenic edited cells. The alternative use of endogenous selectable markers may circumvent this issue (Zhu et al., 2020).

Imidazolinone herbicides inhibit a key enzyme in the plant’s branched chain amino acid biosynthetic pathway, namely, acetolactate synthase (ALS). Many weeds regularly subjected to treatments with these chemicals evolved herbicide-resistant biotypes by obtaining spontaneous point mutations of their *ALS* gene. Those leading to substitutions at the Ala122, Pro197, Ala205, Asp376, Trp574, and Ser653 positions are the most extended (Cross et al., 2015). Taking advantage of this information, imidazolinone-resistant crops have been developed through random-induced mutagenesis and selection, mostly by using single combined amino acid substitutions at positions Ala205, Trp574, and Ser653 (Tan et al., 2005). More recently, using the cytidine base editing system, *ALS* genes from some herbaceous crops in which weed control is required have been mutated to obtain imidazolinone resistance in the field, such as rice (Shimatani et al., 2017), oilseed rape (Cheng et al., 2021), wheat (Zhang et al., 2019), and watermelon (Tian et al., 2018), among others [reviewed by Han and Kim (2019)]. To our knowledge, the only tree crop in which imidazolinone resistance has been introduced by genomic edition is the pear (Malabarba et al., 2021), although base editing has also been tested in apples (Malabarba et al., 2021). Co-edition of the target gene/s of interest and induction of endogenous herbicide resistance could be a valuable methodology to select cells/plants with the desired mutations if regeneration takes place in a proper herbicide-rich medium. We tested this strategy in citrus, (i) by evaluating whether imidazolinone can be used as selection agents for the production of transgenic citrus and (ii) setting up the functionality of cytidine deaminase editing systems to obtain herbicide-resistant edited citrus plants.

## Results

### Evaluation of Imazapyr Toxicity and Influence on Citrus Shoot Regeneration

The impact of different imazapyr (IMZ) doses on shoot proliferation was assessed using epicotyl explants from 6-week-old *in vitro*-grown seedlings of Carrizo citrange (*Citrus sinensis* L. Osb. X *Poncirus trifoliata* L. Raf.). The maximum number of explants developing shoots was found in the non-supplemented condition (0 μM IMZ), and the regeneration response was strongly reduced in the presence of the herbicide at any tested dose (Supplementary Figures 1A,B). At an IMZ dose of 5 μM or higher, the explants did not regenerate any shoots. At 1 μM, less than 0.2% of the explants regenerated shoots, with an average of less than 0.02 shoots per explant, and none of these shoots progressed further. When the herbicide was assayed at a 0.5 μM concentration, only 9.5 ± 2.89% of the explants were able to regenerate shoots, with a medium rate of 0.25 ± 0.02 shoots per explant. Despite at 1 μM the% of explants with shoots was lower than at 0.5 μM, at both conditions, the shoot development was arrested soon from early stages, suggesting that the lower dosage would be enough for *in vitro* culture. A new experiment using these two herbicide concentrations was performed to confirm that shoot development was equally inhibited at 0.5 and 1 μM. Without selection agent, the explants started to shoot in the first week after their transference to light condition increasing the number of shoots per explants along subsequent weeks (Supplementary Figures 1C,D). When IMZ was added to culture media, the shooting ability of the explants was reduced more than 84% and did not increase over the time. The effect of the herbicide was patent from early stages delaying shoot development and avoiding their growth (Supplementary Figure 1E). The percentage of shooting explants and the number of shoots per explant were again slowly higher at 0.5 than at 1 μM (Supplementary Figure 1C) but differences were not statistically significant and shoot development was equally arrested at both doses (Supplementary Figure 1E), hence the former was selected for future experiments.

### Identification of the Citrus Acetolactate Synthase Gene and Generation of Citrange Transgenic Lines to Evaluate Induced Imazapyr Resistance

**FIGURE 1:**
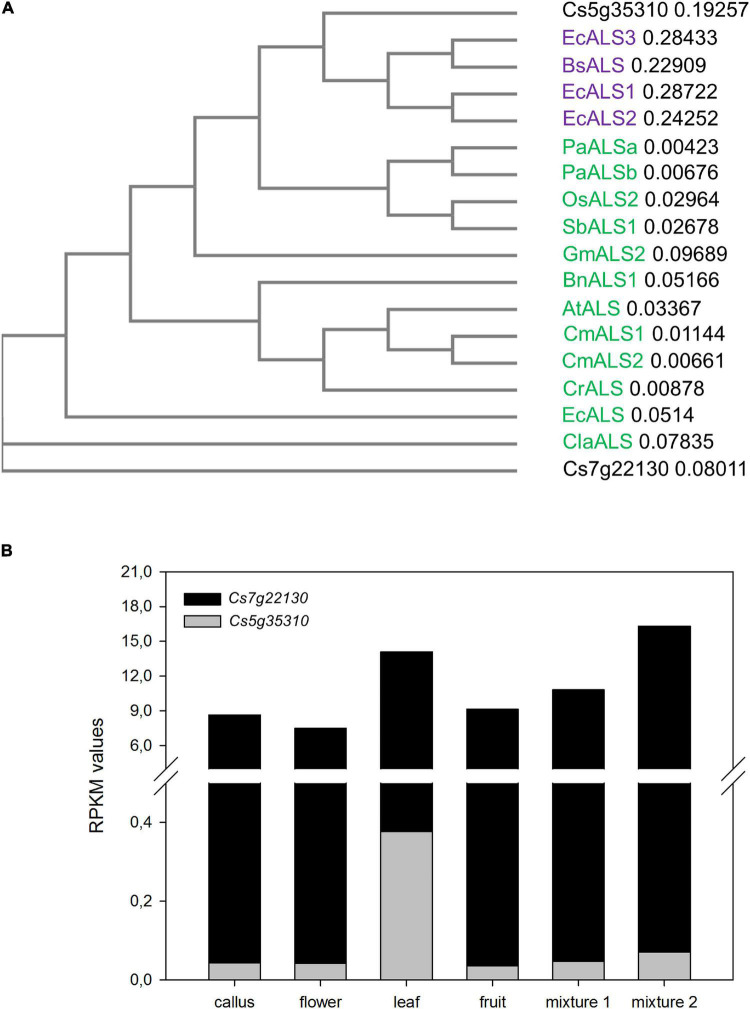
**(A)** Phylogenetic tree of proteins coded by *Cs5g35310* and *Cs7g22130* and selected bacterial [*Escherichia coli* acetolactate synthase 1 (ALS1), ALS2, and ALS3 (WP_032185508, WP_074505494, and WP_115738825, respectively) and *Bacilus subtilis* ALS (WP_129110543)] and plant [*Brassica napus* (XP_013648961), *Camelina microcarpa* ALS1(AAR07632) and ALS2 (AAR06607), *Arabidopsis thaliana* (AAK68759), *Capsella rubella* (XP_006293107), *Erigeron canadensis* ALS (AEE68995), *Glycine max* ALS2 (XP_003545907), *Oryza sativa* ALS2 (NP_001142174), *Sorghum bicolor* ALS1(XP_021315526), *Poa annua* ALSa (ALE27652) and ALSb (ALE27653) and *Citrullus lanatus* ALS (Cla019277)] ALS enzymes. Bacterial and plant enzymes are purple and green lettered, respectively, while predicted citrus ALS enzymes are written in black. **(B)** Reads per kilobase per million mapped reads (RPKM) values in leaf, flower, fruit, and three different mixtures of tissues for *Cs5g35310* and *Cs7g22130*, according to RNA-seq data from Citrus Annotation Project database (CAP; see text footnote 1).

The *Arabidopsis thaliana* ALS protein sequence (AAK68759) was blasted against the Citrus Annotation Project (CAP^1^) database. Two predicted peptides, Cs7g22130 and Cs5g35310, with 89 and 69% identity to *A. thaliana* ALS were found. In both citrus predicted proteins, conserved motifs commonly found in other ALS enzymes, such as the thiamine pyrophosphate-binding enzyme conserved site (IPR000399), domains of thiamine pyrophosphate enzyme (IPR011766, IPR012000, and IPR012001) and ALS large subunit domain (IPR012846, which spans full sequences), were recognized (Supplementary Figure 2). However, such motifs were conserved to a higher extent in Cs7g222130 than in Cs5g35310. When their phylogeny was analyzed in relation to ALS enzymes from other plants and bacteria, Csg535310 clustered with bacterial enzymes (Figure 1A). In addition, *in silico* gene expression analysis of retrieved putative *CsALS* genes showed that *Cs5g35310* transcription was almost negligible[reads per kilobase per million mapped reads (RPKM) 0.03–0.37], while that of *Cs7g22130* was much higher in all analyzed tissues (RPKM 5.61–16.31, Figure 1B). Collectively, this information encouraged us to consider *Cs7g22130* as a good candidate of gene coding for CsALS. Then, its sequence was analyzed to check whether adenosine or cytosine base editors (creating base substitutions of A → G or C → T, respectively) combined with Cas9 with different protospacer adjacent motifs (PAMs) recognition sequences (SpCas9, NGG; SaCas9, NNGRRT; NmCas9, NNNGATT; StCas9, NNAGAAW) were capable of inducing a nucleotide change leading to imidazolinone resistance. The only chance to achieve this goal resulted from the combination of SpCas9 and APOBEC1 cytidine base editor (CBE), which could result in a Ser644Asn amino acid substitution.

Before performing genome edition attempts, the ability of mutant forms of CsALS to confer resistance to IMZ was evaluated *via* transgenesis. To this end, stable *Agrobacterium tumefaciens*-mediated genetic transformation experiments were carried out with a binary plasmid harboring a mutated version of *CsALS*, namely, CsALSm1, coding for a peptide with substitutions Ala196Val, Trp565Leu, and Ser644Asn (Ala205Val, Thr574Leu, and Ser653Asn in *A. thaliana* numbering). A second mutated version of *CsALS* (CsALSm2, harboring Ser644Asn replacement) was also used in *A. tumefaciens*-mediated transformation experiments (Supplementary Figure 3). Epicotyl segments from Carrizo citrange seedlings grown *in vitro* were transformed with both binary constructs and cultured on medium supplemented with IMZ (0.5 μM). For each construct, six independent transgenic lines, checked by PCR for the integrity of the T-DNAs and showing GUS activity in leaves (Supplementary Figure 3), were selected for subsequent evaluations.

### Characterization of Imazapyr Resistance in ALSm1 and ALSm2 Transgenic Citrange Lines

**FIGURE 2:**
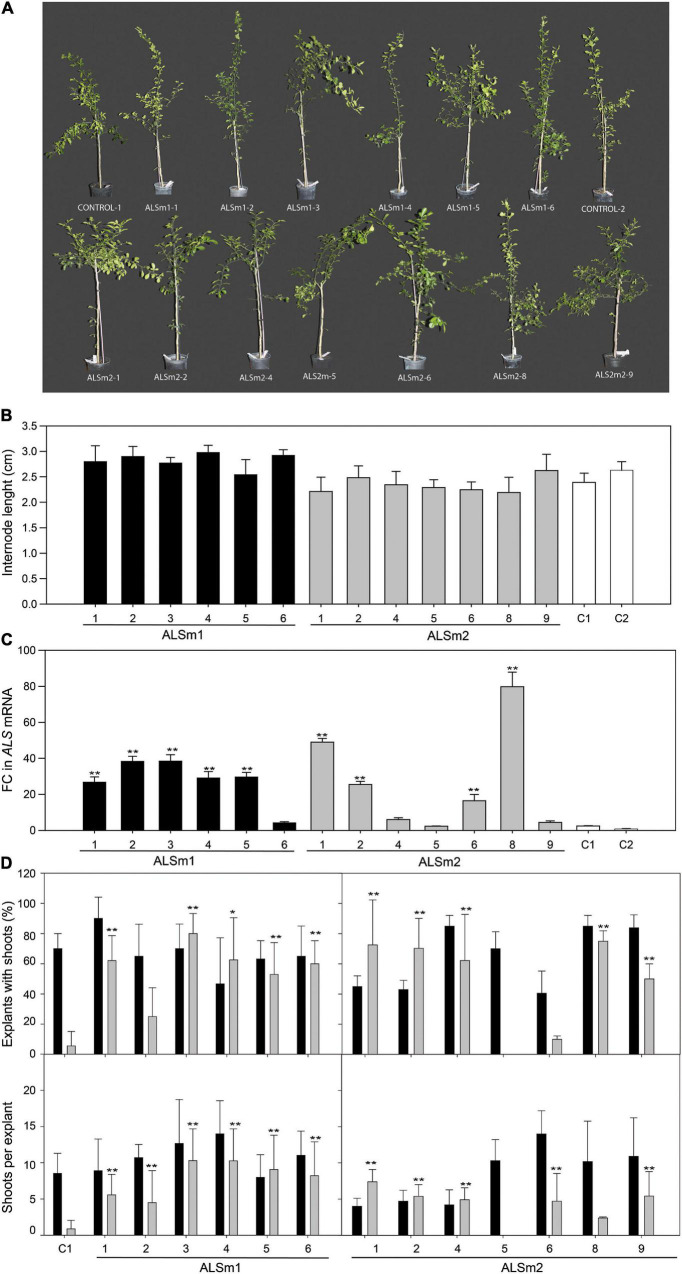
**(A)** Representative pictures of whole plants and **(B)** evaluation of internode length of shoots from ALSm1, ALSm2, and control non-transformed Carrizo citrange lines after 12 months of growth and cultivation in the greenhouse. **(C)** Fold-change (FC) in *ALS* expression between engineered (ALSm1, ALSm2) and non-transformed lines. **(D)** Shooting ability, represented as explants with shoots (top) and shoots per explant (down) of control, ALSm1, and ALSm2 explants in culture medium without selective pressure (black bars) or supplemented with 0.5 imazapyr (gray bars). Bars represent the mean values ± SD of at least three biological replicates. Significant differences in relation to C1 (regenerated control) and ALSm5 (non-transgenic escape) after LSD tests are marked by asterisks (*p < 0.05; **p < 0.01).

As the putative effects of *CsALS* mutations have not been evaluated before, the phenotype of ALSm1 and ALSm2 transgenic lines was evaluated in 1-year-old plants grown in the greenhouse. ALSm1 and ALSm2 trees showed the same architecture as non-transgenic controls as well as comparable shape and leaf size (Figure 2A). Moreover, no yellowing, necrosis, or other symptoms that may be associated with harmful alterations were observed in any of the investigated lines, either transgenic or not (control regenerated shoots). They all showed the same growth patterns, measured as internode length (Figure 2B).

To corroborate that ALSm1 and ALSm2 transgenic lines were regenerated in IMZ selective medium because they acquired resistance to the herbicide, explants from greenhouse-grown transgenic plants were cultured *in vitro* on IMZ-containing medium, and their regeneration was evaluated and compared with that of wild-type non-transgenic control plants (Figure 2C). Line ALSm2-5, which does not harbor any T-DNA insertion (it is an escape from a transformation experiment) (Supplementary Figure 3), was also evaluated as a control. In culture medium without the selection agent, explants from all analyzed lines were able to regenerate shoots at different average percentages (35–90%), but differences in regeneration between lines were not statistically significant, indicating that shoots from all lines displayed similar growth rates (Figure 2D). When IMZ was added to the culture medium, differences in organogenic ability between the non-transformed control, ALSm2-5 and transgenic lines were disparate. In the two controls (C1 and ALSm2-5), the regeneration ability of the explants dropped to 5.55 ± 9.57 and 0%, respectively, while the percentage of regenerants from most ALSm1 and ALSm2 lines did not differ from those generated on non-selective media. Additionally, the number of shoots per explant decreased drastically in both control lines when IMZ was added to the culture medium, whereas in all ALSm1 lines and in most ALSm2 lines, it remained similar under both growing conditions, namely, with or without IMZ in the culture medium. In some ALSm2 lines (ALSm2-6 to ALSm2-9), regeneration was slightly reduced in IMZ-containing medium, but they still produced approximately 2–5 shoots per explant. The much higher number of explants producing shoots and of shoots per explant for ALSm1 and ALSm2 explants in IMZ-containing medium indicated that mutant variants of *ALS* induced resistance to the herbicide in the regenerated shoots because explants from all lines showed similar regeneration ability in non-IMZ-supplemented culture medium.

### *CsALS* Induced Mutations by Genomic Edition in T-DNA-Free Shoots

**FIGURE 3:**
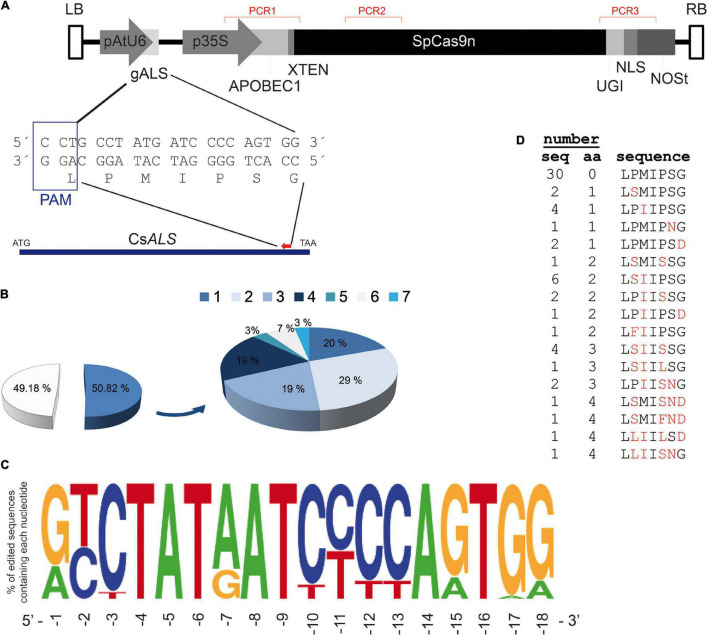
**(A)** Schematic representation of the T-DNA used to edit the endogenous citrus *ALS* gene. LB and RB, left and right T-DNA borders, respectively; pAtU6 and p35S, *Arabidopsis thaliana* polIIIU6 and CaMV 35S promoters, respectively; gALS, gRNA targeting *ALS*; APOBEC1, cytidine deaminase from rat; SpCas9n (SpCas9 D10A nickase mutant); UGI, *Bacillus subtilis* uracil DNA glycosylase inhibitor; NLS, nuclear localization signal of SV40 (simion virus 40) large T antigen; NOSt, nopaline synthase gene terminator sequence. Red-marked regions PCR1, PCR2, and PCR indicate target amplicons to test T-DNA insertion (further details in Supplementary Figure 4). The approximate position of gRNA on the *CsALS* genomic sequence, with its corresponding nucleotides in the gene, is marked by a red triangle. **(B)** Percentage of escapes (49.18%) and edited events (50.82%) from the total of non-transgenic regenerants (288 shoots). Edited shoots presented from one to up to seven C/G→T/A conversions. **(C)** Graphical representation of deamination efficiency found at the different nucleotide positions of the target sequences. Numbers under the sequence indicate nucleotide positions from the PAM sequence. **(D)** Amino acid sequences of the target sequence from non-transgenic regenerant events. The number of events (seq) and amino acid changes (aa, highlighted with red letters in last column) found for each peptide sequence are indicated.

To edit citrus ALS *via* deamination for obtaining a mutant form containing Ser644Asn, substitution, which has been shown to confer resistance to IMZ (Figure 2), a guideRNA (gRNA) targeting C1931 on the reverse DNA strand was designed (gALS, Figure 3A). Included in the deamination window are two other Cs (at positions 1933 and 1934) coding for Gly645. The shoots regenerated after Carrizo citrange epicotyl transformation with *A. tumefaciens* carrying gALS-nCas9-APOBEC1-UGI were analyzed to test T-DNA integration by PCR amplification of three different regions spanning the T-DNA (Figure 3A). From 288 regenerated shoots, 61 not showing amplification products from the T-DNA (Supplementary Figure 4) were further analyzed to investigate gDNA edition, and the rest were considered transgenic and not subjected to more analysis. Approximately 50% of T-DNA-free shoots (31 out of 61) presented C/G→T/A conversions within the deamination window, while the other 30 presented wild-type sequences, which indicated that the latter were likely escapes (Figure 3B). Variable edition efficiencies were found in the different analyzed shoots, ranging from 5 to 28%, being then all regenerated shoots mosaics of edited/non-edited cells. Deamination at two different positions was found in about 29% of the analyzed sequences, while almost 20% of sequences presented deamination at 1, 3, or 4 positions. We found maximum deamination efficiency (58%) at the −7 position counting from the PAM (Figure 3C and Supplementary Table 1), while the efficiency at the target C position (G_–15_ from the PAM), as well as for the other Cs in the strand not paired to the gRNA, was below 30%. C-T conversions on the target strand with efficiencies ranging from 6.45% (C_–3_) to 55% (C_–2_) were also detected.

As a result of deamination at the *CsALS* locus, 16 new sequence variants of the corresponding coded proteins were generated in the 31 edited regenerants (Figure 3D). Near a quarter of the analyzed sequences encoded proteins with single amino acid changes, namely, Pro640Ser, Met641Ile, Ser644Asn, and Gly645Asp (corresponding to AtALS 649, 650, 653, and 654 positions). The remaining edited sequences displayed 2–4 amino acid changes affecting positions 640, 641, 643, 644, and 645.

## Discussion

Globally, citrus are one of the main fruit tree crops in terms of production and cultivated area. This crop is subjected to many biotic and abiotic stresses that compromise their productivity and even their survival. Genetic resistance to different pests and diseases has been achieved by transgenic approaches (Fagoaga et al., 2001; Soler et al., 2012; Rodríguez et al., 2014; Hao et al., 2016; Kobayashi et al., 2017; Sandhu et al., 2019; De Francesco et al., 2020), but any of these new genotypes is been used in the field, probably because of consumers’ rejection to GMOs. Currently HLB, a still incurable citrus disease, is threatening commercial citriculture almost worldwide and it has been proposed that the only chance to fight it is to generate genetically modified resistant cultivars (Yan et al., 2015; McCollum and Baldwin, 2016; NASC, 2018; Cochrane and Shade, 2019; Wang, 2020; Alquezar et al., 2021). By means of transgenesis, different Cas9 enzymes have been already proved to be able to generate edited knockout citrus mutants (Jia et al., 2017a,b, 2019; Peng et al., 2017; LeBlanc et al., 2018; Jia and Wang, 2020). In herbaceous crops, edited non-transgenic cultivars are usually developed by, after getting homozygous edited lines, performing sexual crosses until removing the T-DNA containing selection markers and CRISPR/Cas-associated transcriptional units. In tree crops, a similar breeding program to remove foreign DNA is unapproachable because of the great investment of time and money that it would entail. Launching a system to use CRISPR/Cas technology without the introduction of foreign DNA would allow obtaining resistant non-transgenic tree cultivars in an economic and time-affordable manner.

To minimize the resources directed to recover edited non-transgenic tree plants is key to establish a selection system that, during the *in vitro* culture stage, minimizes the amount of plant material to assess and favor the survival of edited cells/shoots only. In weeds, it is well-known that resistance to pyrimidil benzoates, imidazolinones, triazolinones, sulfonylureas, and triazolopyrimidines herbicides can arose by spontaneous mutations in *ALS* gene (Tranel et al., 2021). In last years, this information is being exploited to control weeds by developing new herbicide-resistant herbaceous crops via editing technology (Han and Kim, 2019). Very recently, the transgenic lines of pear, in which the endogenous gene was base edited by APOBEC1 cytidine deaminase, acquired resistance to chlorsulfuron (Malabarba et al., 2021). The use of endogenous selection genes is useful to develop cisgenic or intragenic cultivars, better perceived by consumers than transgenic ones (Malabarba et al., 2021). These genes could also be useful to generate tree edited crops free of foreign DNA. To explore these possibilities, we decided to test the potential of *CsALS* and the imidazolinone IMZ as selection agent in citrus transformation experiments, for which kanamycin or reporter genes are usually employed (Jia and Wang, 2014, 2020; Jia et al., 2017a,b, 2019; Peng et al., 2017; LeBlanc et al., 2018; Huang et al., 2020).

On the one hand, by dose-response *in vitro* culture experiments, we confirmed that the herbicide IMZ could restrict shoot regeneration from citrus explants. By the other hand, we identified by homology search two citrus genes, *Cs5g35310* and *Cs7g22130*, putatively coding for ALS. The last coded for a peptide that, besides having conserved ALS-motifs, claded closer to ALS enzymes from dicotyledonous plants. In addition, *Cs7g22130* envisaged higher transcription level than that of *Cs5g35310*, and thus it is more coherent with that expected for a gene involved in primary metabolite biosynthesis. These results indicated that *Cs7g22130* most probably codes for CsALS. We then analyzed its genomic sequence searching for point mutations that could be generated by CRISPR/Cas9 base editors. Inducing the most extended ALS mutation leading to herbicide resistance (Cross et al., 2015; Tranel et al., 2021), namely Trp574Leu (standardized to the *A. thaliana* sequence), was unapproachable by base editors in *CsALS*, where this aminoacidic position is coded by TGG triplet and any G → A conversion would lead to a stop codon. *Amaranthus* spp. (*tuberculatus*, *hybridus*, and *palmeri*), *Setaria viridis*, *Galium spurium*, *Bromus tectorum*, and *Avena fatua* natural mutants harboring Ser653Asn mutation (standardized to the *A. thaliana* sequence) are highly resistant to imidazolinones (Tranel et al., 2021), so we decided to attempt this goal by directing SpCas9-APOBEC1 CBE to induce CsALS-G1931A replacement.

Before performing genome edition attempts, the transgenic citrus plants were generated to evaluate whether the Ser644Asn mutation in CsALS (CsALSm2) conferred resistance to the herbicide IMZ. To ensure that IMZ-resistant transgenic lines were obtained, a second mutated version of *CsALS* (CsALSm1), harboring besides Ser644Asn the extended replacements Ala196Val and Trp565Leu was also used. The former, noted as Ala205Val in AtALS, is not extended as the latter in natural mutants, but it has been reported in *Xanthium*, *Helianthus*, *Amaranthus*, *Solanum*, and *Conyza* species (Tranel et al., 2021). The changes in ALS could affect plant growth and fitness depending on both the mutation/s and plant species. For example, the Trp574Leu substitution negatively altered green amaranth development and fitness (Tardif et al., 2006) but not those of ryegrass or wild radish plants (Yu et al., 2010; Li et al., 2013). Phenotypically, CsALSm1 and CsALSm2 trees were indistinguishable from wild-type control plants, being the only remarkable differences a higher *ALS* transcription level (due to transgene expression under control of CaMV35S promoter) and the acquired ability to regenerate in IMZ-containing medium. Although transgenic lines may still harbor wild-type alleles for *CsALS*, which will not be present in genome edited homozygous lines, these preliminary results indicate that the selected ALS mutations would most likely not affect plant fitness. Consequently, mutated versions of the citrus *ALS* gene and IMZ can be used instead of kanamycin and *nptII* to select transgenic citrus shoots, opening the possibility, non-existent until now, to develop new intra- or cisgenic cultivars, as recently proposed for pear and apple trees (Malabarba et al., 2021).

Although, in comparison with transgenic cultivars, the use of cis/intragenic strategies would already mean an improvement in the perception of consumers, their total acceptance would be probably achieved through the development of edited plants free of foreign DNA. Citrus genome edition has been successfully accomplished using Cas9 from different sources. However, the recovery of edited plants still relied on kanamycin-supplemented medium and/or in the use of visual markers to select transgenic plants harboring Cas9-containing cassettes (Jia et al., 2017a,b, 2019; Peng et al., 2017; Zhang et al., 2017; Jia and Wang, 2020). Elimination of transgenes from edited lines is practically unapproachable in commercial citrus genotypes because, in addition to their complex reproductive characteristics, sexual crosses (if attainable) to eliminate foreign DNA maintaining edited regions could take decades and most likely would alter their elite features. Editing the *ALS* gene and herbicide-mediated selection of regenerating shoots would potentially allow the recovery of transgene-free edited citrus plants because before T-DNA integration its transcriptional units are expressed transitorily in the target nucleus. Only a small ratio of T-DNA strands is efficiently integrated while most of them are just transiently expressed and then degraded by cell nucleases (Komor et al., 2016). In apple, the transient expression of Cas9-module from T-DNA led to the recovery of edited non-transgenic at a frequency of 0.4% (Veillet et al., 2019). By targeting a CBE to *CsALS* to induce Ser644Asn replacement and using IMZ as selection agent, the percentage of regenerated shoots that were edited and T-DNA-free was close to 11%, very similar to those obtained in potato and tomato (10 and 13%, respectively) by targeting deamination at nucleotides coding *ALS* Pro197 and using chlorsulfuron as a selective agent (Zhang et al., 2017). As usual in transgenic and edited tree crops, such as apple, pear, banana, grapevine, and citrus (Malabarba et al., 2021), all the obtained non-transgenic edited shoots were chimeras, being the maximum of edited cells in a shoot of 28%. Recently, it has been reported that in *Rosaceae* spp., such chimerism can be greatly reduced by performing an additional step of *in vitro* adventitious regeneration under selection pressure (Malabarba et al., 2021), a technique that could also be used to improve the% of edited cells in T-DNA-free citrus regenerants. Other aspects of this methodology also have to be further optimized. For example, we did not found maximum deamination efficiency between positions -12 and -17 counting from the PAM, as expected using XTEN between SpCas9 and APOBEC1 (Komor et al., 2016; Veillet et al., 2019), but using deaminase mutants with altered deamination windows may solve this issue (Kim et al., 2017). Besides, unexpectedly because we were using a nickase version of SpCas9 (D10A) to selectively cut the target DNA strand (Veillet et al., 2019), we detected C-T conversions on it. Recently, it has been reported that base editors retain some Cas9-independent deamination activity, mainly in actively transcribed regions of the genome (Doman et al., 2020). In addition, targeted deaminases work more efficiently if the target DNA is transcriptionally active (Yang et al., 2016). We hypothesized that because of IMZ supplementation on selection medium, *CsALS* may be highly transcribed, as both target and non-target strands are prone to deamination. In any case, all regenerated non-transgenic edited plant harbor 1 to 4 aminoacid changes at Pro640, Met641, Ser644, or/and Gly645 (corresponding to AtALS 649, 650, 653, or/and 654 positions) that for sure or most probably confer IMZ resistance. For example, substitution of Ser653 (standardized to the *A. thaliana* sequence) for Thr, Asn, or Ile renders imidazolinone resistance in different spontaneous weed mutants (Tranel et al., 2021) while Gly654Asp change confers imidazolinone resistance to *S. viridis* mutants (Laplante et al., 2009), and to edited wheat lines (Zhang et al., 2019). For both Ser653 and Gly654 (*A. thaliana* numbering), resistance to imidazolinones derives from non-conservative amino acid substitutions that alter the herbicide binding site (Laplante et al., 2009; Rojano-Delgado et al., 2019). According to the *A. thaliana* protein model (UniProtKB–P17597), Pro640 and Met641 are located in the same loop as Ser563 and Gly654, and non-conservative substitutions of these amino acids most likely alter the IMZ binding site, thus leading to herbicide resistance. Moreover, the generation of plants harboring these mutations under IMZ selection suggests that all of these (individually or in combination) confer herbicide resistance.

In conclusion, we have developed a strategy to recover T-DNA-free edited citrus plants by combining the use of a base editor system and IMZ selection. The efficiency of the herbicide as a selection agent is shown using a transgenesis approach, leading to the recovery of T-DNA harboring lines with good efficiency. CRISPR-induced deamination of the *ALS* gene endows regenerant shoots with the ability to grow in IMZ-selective medium. By analyzing non-transgenic shoots regenerated from edition experiments, we observed that the transient expression of Cas9-APOBEC1 system components from T-DNA induces citrus gDNA edition during *A. tumefaciens* cocultivation at high frequency. Our future work will be directed to further optimize the edition process during the transitory expression of *A. tumefaciens* T-DNA and/or to improve the already used or to introduce new *in vitro* culture stages in order to increase the percentage of edited cells in regenerated plants. The generation of edited shoots free of foreign DNA indicates that the combined use of base deamination systems targeting gene/s of interest and *ALS* as a co-editing marker can be used to recover non-transgenic edited new citrus cultivars. To our knowledge, this is the first report showing that this is an affordable strategy to obtain non-transgenic edited tree crops.

## Materials and Methods

### Citrus Acetolactate Synthase Gene Cloning and Site-Directed Mutagenesis

Total RNA (1 μg) was extracted from the peel of mature sweet orange (Clonorchis *sinensis* L. Osb. cv. Pinneaple) and Carrizo citrange (*Citrus* X *sinensis* L. Osb. X *Poncirus trifoliata* L. Raf.) fruits. The RNA was treated with DNase using an RNeasy mini kit (Qiagen) and reverse transcribed using 200 U of SuperScript II Reverse Transcriptase (Invitrogen, Karlsruhe, Germany) and 500 ng of oligodT primer. Then, primers B226 and B227 (Supplementary Table 2) were employed to PCR-amplify the full-length Cs7g22130 coding sequence from fruit cDNAs using CloneAmp HiFi polymerase (Clontech, Takara Bio Europe SAS, Saint-Germain-en-Laye, France) following the manufacturer’s instructions and 1 μL of cDNA as a template. The only single amplicon obtained for each genotype was purified using E.Z.N.A. Cycle pure kit (OMEGA, Norcross, United States), cloned into pJET1.2/blunt (Thermo Fisher Scientific, Waltham, United States), and used to transform *Escherichia coli* DH5α. Four independent clones per genotype were sequenced, showing 100 and 99.85% identity of sweet orange and citrange with the Cs7g22130 amino acid predicted sequence, respectively.

Two mutated versions of *ALS* were generated by introducing nucleotide changes on the cloned wild-type gene from sweet orange (*CsALS*). One mutated version (CsALSm1) coded for a protein with Ala196Val, WTrp565Leu, and Ser644Asn amino acid substitutions, while the second version (CsAHASm2) harbored only the Ser644Asn change. *CsALSm1* was generated by PCR amplification of pJET-CsALS with primer pairs B234/pJET-F, B232/B235, B230/B233, B228/B231, and B229/pJET-R (Supplementary Table 2) using CloneAmp HiFi polymerase (Clontech, Takara Bio Europe SAS, Saint-Germain-en-Laye, France). An equimolar mix of PCR products was prepared and used as a DNA target for CloneAmp HiFi polymerase for seven amplification cycles. Just before the 8th amplification cycle, primers B226 and B227 were added to the reaction and led to 25 more cycles. Full-length amplicons were isolated from agarose gels, cloned on pJET, and sequenced. *CsALSm2* was generated in the same way but using B234/pJET-F and B229/pJET-R resulting amplicons as DNA targets for subsequent amplification.

### Constructs Generation

The *CsALSm1* and *CsALSm2* were excised from the pJET plasmid by *Bsm*BI digestion and gel-purified using an E.Z.N.A. Cycle pure kit (Omega, Norcross, United States). The pUPD plasmid and 40 ng of each purified digestion were used to perform a *Bsm*BI GoldenBraid (GB) reaction as previously described (Sarrion-Perdigones et al., 2013). pUPD-CsAHASm1 and pUPD-AHASm2 were subjected to subsequent GB reaction with *Bsa*I and GB0030, GB0037 and pDGB3a2 plasmids to generate binary vectors harboring the 35S promoter:AHAS(m1 or m2):NOS terminator within the T-DNA. These vectors were used in further GB reactions to introduce a 35S:GUSintron:NOSt cassette. pDGB3Ω2-CsAHASm1 (35S:CsAHASm1:NOSt_35S:GUSint:NOSt) and pDGB3Ω2-CsAHASm2 (35S:CsAHASm2:NOSt_35S:GUSint:NOSt) binary plasmids were used for citrus genetic transformation experiments (Supplementary Figure 3). T4 DNA ligase from Promega (M180B), *Bsa*I from NEB (R0535S), and *Bsm*BI from Fermentas (ER0451) were used.

For genomic edition experiments targeting *CsALS*, a gRNA tiled to a protospacer-adjacent motif (AGG) located on the reverse DNA strand was designed manually (5′-CCACTGGGGATCATAGGC-3′-). gRNA was shortened to 18 nct because it has been proposed that truncated gRNAs may narrow deamination window (Kim et al., 2017). On- and off-target efficiencies were confirmed on Benchling (Cloud-Based Informatics Platform for Life Sciences R&D^2^) using the sweet orange genome as a reference (GenBank GCA_000317415.1). The Benchling software confirmed that nucleotides encoding S and G were located in the editing window of cytidine deaminase (Supplementary Table 3). Off-target efficiencies calculated on the basis of 20 nucleotide gRNA were 1.5 for scaffold_0123:-544151 and equal to or lower than 0.2 for the other six chromosomal regions (Supplementary Table 4). A cassette harboring APOBEC1 cytidine deaminase and uracil DNA glycosylase inhibitor (UGI) fused using XTEN linker to N-terminus of a nickase form of SpCas9 (nSpCas9 D10A) was isolated from plasmid pXSE901BG (#91714 Addgene) by PCR-amplification using primers (MU20 and MU21) and cloned by standard GB procedures in pDGB3α2 *Bsa*I restriction site. A target adaptor containing *gALS* was prepared by natural annealing of primers B413 and B414 (Supplementary Table 2) and cloned on pDGB3α1 in combination with the AtU6–26 promoter and a total RNA (tRNA) scaffold (GB1001 and GB2245, respectively) using standard GB procedures. Both expression cassettes from pDGB3α plasmids were finally combined in pDGB3Ω1 and used for citrus transformation.

### Dose–Response Shoot Regeneration Experiments

The seeds from Carrizo citrange were coat-stripped, soaked with sodium hypochlorite (0.4%)-Tween-20 (0.1%) solution for 10 min, rinsed with autoclaved double distilled water four times in a laminar flow hood, and sown in culture tubes containing 40 mL of Murashige & Skoog (MS) medium (containing 0.8% agar and 3% sucrose). The tubes were maintained for 3 weeks in darkness and for an additional 3 weeks under a photoperiod of 2,000 lux for 16/8 h, both at 26°C and 40% RH. Epicotyl segments (approximately 1 cm length) from seedlings grown *in vitro* were cultured on MS medium supplemented with 0.2 mg L^–1^ thiamine-HCl, 1 mg L^–1^ pyridoxine-HCl, 1 mg L^–1^ nicotinic acid, 100 mg L^–1^ myo-inositol, 30 g L^–1^ sucrose, 3 mg L^–1^ BAP, 125 mg L^–1^ vancomicin, 250 mg L^–1^ cefotaxim, 7.9 g L^–1^ agar, pH 5.8 plus different IMZ doses, including 0 and 0.4–30 μM concentrations of the selection agent.

Shoot regeneration was assessed by counting the average number of explants with shoots and the number of shoots per explant. Three replicates of 80 explants were used for each treatment, and regeneration was evaluated after 6 weeks of exposure to IMZ.

To test the herbicide resistance of ALSm1 and ALSm2 whole plants, three independent branches from each line were excised, surface-sterilized, and cut into 1-cm length internode explants that were placed on selective culture medium containing 0.5 μM IMZ. Culture conditions were the same as those described above for wild-type explants. Experiments were conducted in triplicate with a minimum of 80 explants each. Two-way ANOVA and the means least significant difference (LSD) test at the 5% *p*-level were used for statistical analysis using STATGRAPHICS Centurion XVII software (version 17.2.00).

### Citrus Genetic Transformation

For genetic transformation, epicotyl segments from Carrizo citrange seedlings prepared as for shoot regeneration experiments were used. In brief, epicotyl explants were incubated with a bacterial suspension of the *A. tumefaciens* EHA105 strain previously transformed with pDGB3Ω2-CsAHASm1 or pDGB3Ω2-CsAHASm2. After 3 d of cocultivation, the explants were transferred to selection medium containing IMZ (0.5 μM) as a selection agent and maintained in the dark for 4 weeks at 26°C and 40% RH. The explants were then transferred to the same temperature and RH conditions but with a 16-h photoperiod and subcultured in fresh medium every 4 weeks.

The genomic DNA from regenerated shoots was extracted using the cetyl trimethyl ammonium bromide (CTAB) method, and the presence of different T-DNA regions was confirmed by PCR using FIREpol (Solis byodine) polymerase and primer pairs B13R/B232, B229/35S finalF, and GUSup/GUSdown for pDGB3O2-CsALSm1 and pDGB3O2-CsALSm2 (Supplementary Table 2 and Supplementary Figure 3). All PCR-positive shoots were shoot-tip grafted *in vitro* onto Troyer citrange seedlings. Approximately 3 months later, the plantlets were grafted in a greenhouse onto 5-month-old Rangpur lime (*Citrus limonia* Osb.) rootstocks potted in coconut fiber. The expression of the *uid* transgene in greenhouse-grown plants was checked by GUS assays performed by overnight incubation of leaf sections at 37°C in 2 mM X-Gluc solution as described previously.

### Quantitative Real Time–PCR Analysis

The total RNA extraction from new flushes and DNase treatment were performed using the RNeasy minikit (Qiagen, Barcelona, Spain). cDNA synthesis and quantitative real-time PCR procedures were performed as described by Carmona et al. (2017). Primers B227 and B230 (Supplementary Table 2) were used to investigate *ALS* expression. Citrus *UPL7* and *GAPC2* were used as housekeeping reference genes. The expression level in shoots of non-transformed control lines was set to 1, and the remaining values referred to it. Values are presented as the mean of at least three independent analyses ± SD.

### Citrus Genomic Edition Experiments and Data Analysis

The Carrizo citrange seedlings were cut in fragments of about 1 cm length and incubated for 10 min with a bacterial suspension of *A. tumefaciens* EHA105 harboring pDGB3O1-gALS-nCas9-APOBEC1-UGI plasmid (Figure 3A), as usually done in citrus genetic transformation experiments. Epicotyl explants were then cultured 3 days on coculture media and darkness condition (26^°^C, 40% RH) and subsequently on 0.5 μM of IMZ-supplemented selection media and light:dark (16:8 h, 70 mE m^–2^ s^–1^) photoperiod at 26^°^C and 40% RH. After 3–4 weeks of culture in this condition, the explants started to shoot. Each individual shoot was excised from the explant and grafted *in vitro* on etiolated two-to-three-old Carrizo citrange seedlings. Before grafting, a small portion of each shoot was frozen in liquid N_2_ and stored at -80^°^C until genomic DNA extraction using CTAB-method. gDNA from each shoot was analyzed for T-DNA integration and the presence of genomic editions. Integration of T-DNA in regenerated shoots was checked by PCR amplification using a set of three primer pairs that cover three different regions of the nCas9-APOBEC1-UGI expression module (Supplementary Figure 4 and Supplementary Table 2). Then, for each shoot, three different PCRs were performed with Firepol polymerase (Biotools) following manufacturer instructions and using 0.25 mM of each primer pair (35S final F and B596 for PCR1; B595 and B600 for PCR2; and B421 and B422 for PCR3, as named in Supplementary Figure 4). Cycling conditions were an initial denaturation of 5 min at 95^°^C, followed by 35 cycles of 30 s 95^°^C/30 s 56^°^C/50 s 72^°^C and a final elongation step of 5 min at 72^°^C. PCR products from these reaction were visualized on a 1% agarose gel supplemented with RedSafe™ nucleic acid staining solution at 1X final concentration. Genomic DNA was extracted and PCR-analyzed for T-DNA insertion from all regenerated shoots from three independent experiments. All gDNAs from non-transgenic shoots were further investigated to analyze genome modifications of endogenous Cc*ALS* target sequence. With this purpose, 1 mL of each T-DNA-free gDNA was used as template to perform a PCR using Phusion High-Fidelity DNA polymerase (Thermo Scientific, Strasbourg, France) as manufacturer indicates and primers B232 and B599 at a final concentration of 0.5 mM (Supplementary Table 2). Cycling conditions were 30 s at 98^°^C followed by 25 cycles of 10 s 98^°^C/10 s 56^°^C/20 s 72^°^C and a final extension step of 5 min at 72^°^C. Amplicons were purified with E.Z.N.A. Cycle pure kit (Omega Bio-tek, Georgia, United States) and subjected to Sanger sequencing with primer B232 in the DNA sequencing facility of the instituto de biología molecular y celular de plantas (IBMCP). Changes (C-T conversions) in the sequences were evaluated directly from applied biosystems (ABI) 3130 XL sequencer AB1 output files using EditR (Kluesner et al., 2018), and the *p*-value cutoff was set to 0.01. According to the literature, editing levels lower than 5% were considered background (Agudelo et al., 2020).

## Data Availability Statement

The original contributions presented in the study are included in the article/Supplementary Material, further inquiries can be directed to the corresponding author.

## Author Contributions

LP conceived the study. BA, LC, and SB performed citrus transformation and edition experiments. BA did data analysis. BA and AG did manuscript draft writing. All authors discussed the results and contributed to the final version of the manuscript.

## Conflict of Interest

The authors declare that the research was conducted in the absence of any commercial or financial relationships that could be construed as a potential conflict of interest.

## Publisher’s Note

All claims expressed in this article are solely those of the authors and do not necessarily represent those of their affiliated organizations, or those of the publisher, the editors and the reviewers. Any product that may be evaluated in this article, or claim that may be made by its manufacturer, is not guaranteed or endorsed by the publisher.

## Footnotes

1
http://citrus.hzau.edu.cn/orange/

2
https://benchling.com

## Supplementary Material

Supplementary Figure 1 **(A–C)** Effect of different imazapyr concentrations on shoot regeneration in Carrizo citrange explants. There were a minimum of 80 explants per treatment, and each treatment was replicated three times. Shooting ability was evaluated 3 weeks (day 21 or 22) after transferring explants to light condition. In **(C)**, an additional evaluation was performed 8 days after light transfer. **(D)** Representative photographs of Carrizo citrange explants at different imazapyr assayed concentrations. **(E)** Representative photographs of regenerated shoots from Carrizo citrange explants maintained in culture media supplemented with imazapyr at different concentrations (0, 0.5 or 1 μM) 8 and 22 days after their transference to light conditions.

Supplementary Figure 2 Amino acid sequence alignment of Cs5g35310 and Cs7g22130 with four and 13 different acetolactate synthase (ALS) proteins from bacteria and plants, respectively. The alignment was generated with Clustal Omega. Residues are color coded according to their conservancy: white letters indicate fully conserved residues, while those marked in dark and light gray present less similar properties with scores greater and lower than 0.5 on the protospacer adjacent motif (PAM) 250 matrix, respectively. Characteristic motifs present in ALS enzymes, such as the TPP-binding enzyme conserved site (IPR000399), domains of thiamine pyrophosphate enzyme (IPR011766, IPR012000, and IPR012001) and ALS large subunit domain (IPR012846, which spans full sequences), are indicated.

Supplementary Figure 3 **(A)** T-DNAs used to generate transgenic imazapyr-resistant lines. Schematic diagram of the T-DNA regions of the pDGB3O2 vectors containing mutated versions of *CsALS* (ALSm1 and ALSm2) used for citrus transformation. LB and RB, left and right T-DNA borders, respectively; p35S, CaMV 35S promoter; *ALS*, mutated versions of *CsALS* CDS. Nucleotide changes in ALSm2 and ALSm1 are indicated; GUSint, β-glucuronidase (*uid*A) gene spanned by an intron; T, terminator region of nopaline synthase gene; KanR, neomycin phosphotransferase II (*nptII*) selectable marker gene conferring kanamycin resistance; pNOS, nopaline synthase gene promoter sequence. The transcription orientation for each cassette is indicated by arrows on the promoter sequence. The approximate positions of the primers used to test T-DNA insertion are shown. **(B)** Confirmation of T-DNA integration by PCR analysis with primers B13R/B232 (PCR1), B229/35S final F (PCR2), and GUSup/GUSdown (PCR3); -, negative PCR control; M, 1 kb DNA molecular marker from Invitrogen. **(C)** Representative glucuronidase (GUS) staining patterns exhibited by leaves from ALS1 and ALS2 transgenic lines and two control plants. All leaves were punched to facilitate substrate infiltration.

Supplementary Figure 4 Identification of T-DNA-free regenerants using three primer pairs. **(A)** Schematic representation of the T-DNA used for *Agrobacterium tumefaciens* transformation experiments. Regions amplified by the different pairs of primers are marked by red lines: PCR1 (35S finalF, B596), PCR2 (B595, B600), and PCR3 (B421, B422). Primer sequences are detailed in Supplementary Table 2. **(B)** Representative results of PCR analysis from 192 regenerant shoots. A 1 kb marker (M) from Invitrogen was loaded on the first lane of each gel row. Identified regenerants without T-DNA insertions are marked with arrows.
