# Supplementary figures and images for: Generation of Transfer-DNA-Free Base-Edited Citrus Plants
Source: Front Plant Sci. 2022 Mar 15;13:835282. doi: 10.3389/fpls.2022.835282 (PMC8965368; doi:10.3389/fpls.2022.835282)

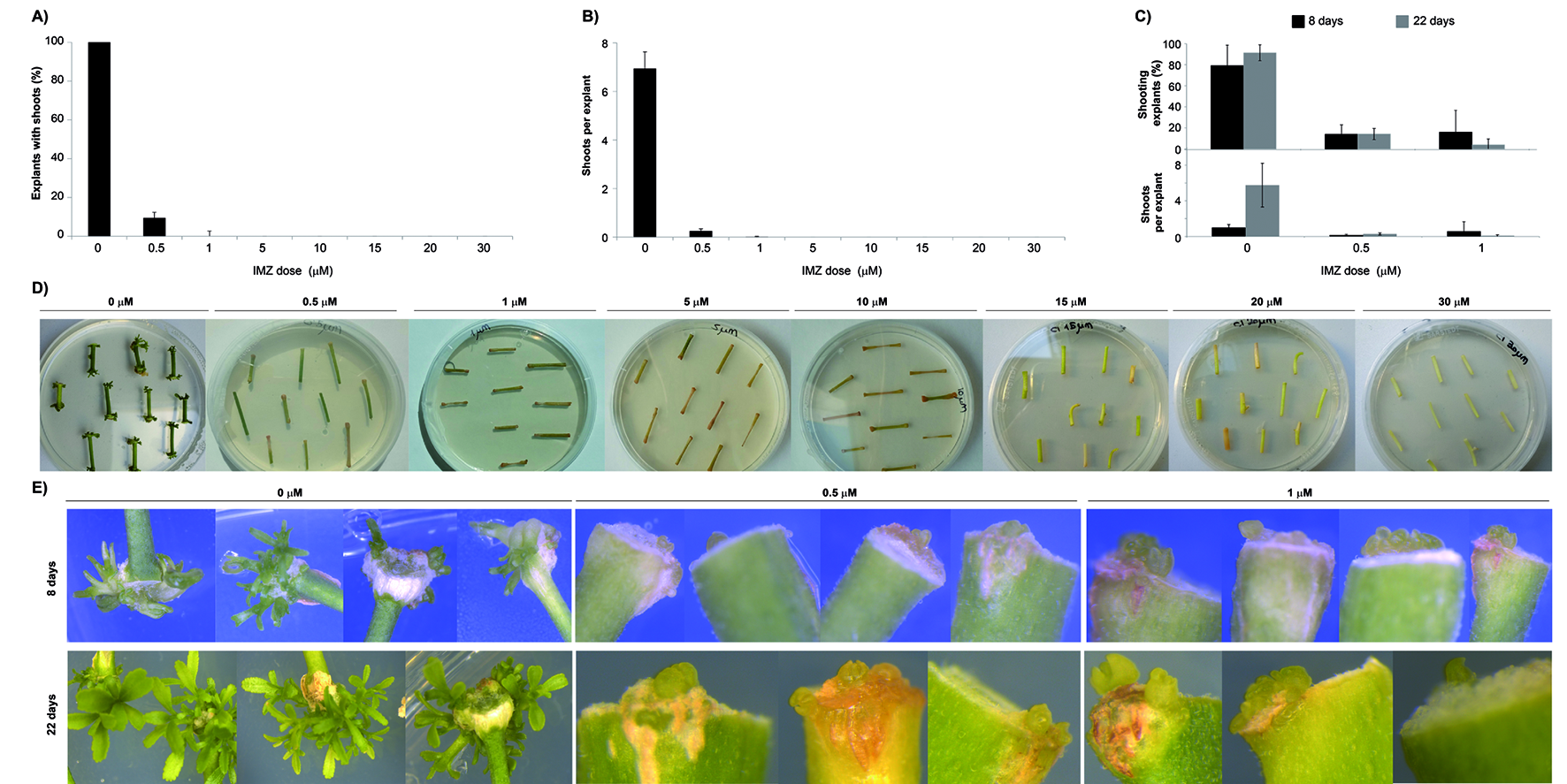

Supplement: Supplementary Figure 1 — (A–C) Effect of different imazapyr concentrations on shoot regeneration in Carrizo citrange explants. There were a minimum of 80 explants per treatment, and each treatment was replicated three times. Shooting ability was evaluated 3 weeks (day 21 or 22) after transferring explants to light condition. In (C), an additional evaluation was performed 8 days after light transfer. (D) Representative photographs of Carrizo citrange explants at different imazapyr assayed concentrations. (E) Representative photographs of regenerated shoots from Carrizo citrange explants maintained in culture media supplemented with imazapyr at different concentrations (0, 0.5 or 1 μM) 8 and 22 days after their transference to light conditions. [file Image_1.TIF]

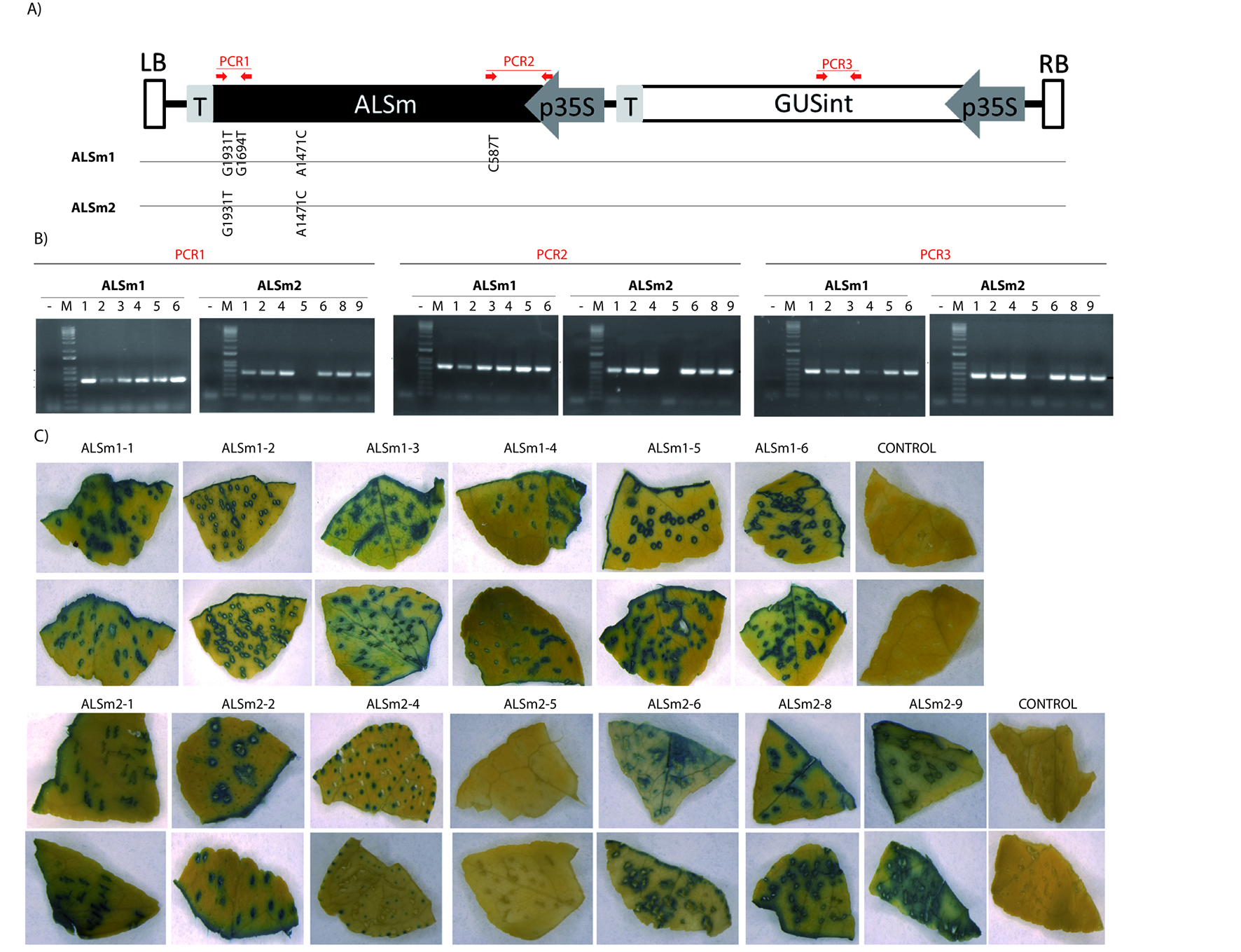

Supplement: Supplementary Figure 3 — (A) T-DNAs used to generate transgenic imazapyr-resistant lines. Schematic diagram of the T-DNA regions of the pDGB3O2 vectors containing mutated versions of CsALS (ALSm1 and ALSm2) used for citrus transformation. LB and RB, left and right T-DNA borders, respectively; p35S, CaMV 35S promoter; ALS, mutated versions of CsALS CDS. Nucleotide changes in ALSm2 and ALSm1 are indicated; GUSint, β-glucuronidase (uidA) gene spanned by an intron; T, terminator region of nopaline synthase gene; KanR, neomycin phosphotransferase II (nptII) selectable marker gene conferring kanamycin resistance; pNOS, nopaline synthase gene promoter sequence. The transcription orientation for each cassette is indicated by arrows on the promoter sequence. The approximate positions of the primers used to test T-DNA insertion are shown. (B) Confirmation of T-DNA integration by PCR analysis with primers B13R/B232 (PCR1), B229/35S final F (PCR2), and GUSup/GUSdown (PCR3); -, negative PCR control; M, 1 kb DNA molecular marker from Invitrogen. (C) Representative glucuronidase (GUS) staining patterns exhibited by leaves from ALS1 and ALS2 transgenic lines and two control plants. All leaves were punched to facilitate substrate infiltration. [file Image_3.TIF]

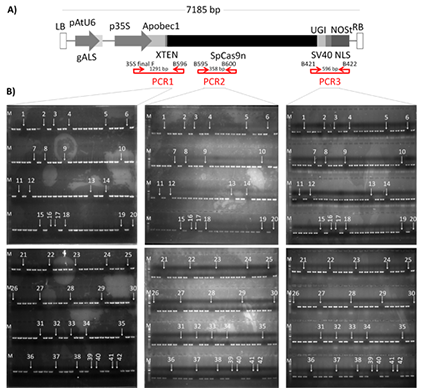

Supplement: Supplementary Figure 4 — Identification of T-DNA-free regenerants using three primer pairs. (A) Schematic representation of the T-DNA used for Agrobacterium tumefaciens transformation experiments. Regions amplified by the different pairs of primers are marked by red lines: PCR1 (35S finalF, B596), PCR2 (B595, B600), and PCR3 (B421, B422). Primer sequences are detailed in Supplementary Table 2. (B) Representative results of PCR analysis from 192 regenerant shoots. A 1 kb marker (M) from Invitrogen was loaded on the first lane of each gel row. Identified regenerants without T-DNA insertions are marked with arrows. [file Image_4.TIF]
