# Supplementary material for: Generation of Transfer-DNA-Free Base-Edited Citrus Plants
Source: Front Plant Sci. 2022 Mar 15;13:835282. doi: 10.3389/fpls.2022.835282 (PMC8965368; doi:10.3389/fpls.2022.835282)
Supplement: Supplementary Figure 2 — Amino acid sequence alignment of Cs5g35310 and Cs7g22130 with four and 13 different acetolactate synthase (ALS) proteins from bacteria and plants, respectively. The alignment was generated with Clustal Omega. Residues are color coded according to their conservancy: white letters indicate fully conserved residues, while those marked in dark and light gray present less similar properties with scores greater and lower than 0.5 on the protospacer adjacent motif (PAM) 250 matrix, respectively. Characteristic motifs present in ALS enzymes, such as the TPP-binding enzyme conserved site (IPR000399), domains of thiamine pyrophosphate enzyme (IPR011766, IPR012000, and IPR012001) and ALS large subunit domain (IPR012846, which spans full sequences), are indicated. [file Image_2.PDF]

```
                                                                                                                    ____IPR12001___
Cs5g35310     MA-----------TTL--SPPFIFN----TPKSPPTISRPLMPLPYSYN--------RIK-----KPSLHVTN-----NTLNIPTSTAPFQQTDFASRFAPDKPRKGADIIVEALERQGV 85
PaALSa        --MATATSTA---VAI--SG--------ATSALPKPSLPRHLPAS----------RRAL-A----------ATRIRCSTV-S---PSPAPPATALRPWGPTEPRKGADILVEALERCGI  79
PaALSb        MATATATSTA---VAI--SG--------ATSALPKPSLPRHLPAW----------RRAALAA----------ATRIRCSTVSS---PSPAPPATALRPWGPTEPRKGADILVEALERCGI 84
OsALS2        --MATAATAA---AAL--TG--------ATTATPKSRRRAHHLAT----------RRAL-------------AAPIRCSALSRA--TPTAPPATPLRPWGPNEPRKGSDILVEALERCGV 80
SbALS1        -MATTAAAAA---AAL--AG--------ATTAAPKARRRAHLLAA----------RRAL------------AAPIRCSAAPPATLTVTAPPATPLRPWGPTDPRKGADILVEALERCGV  83
GmALS2        MA-----------ATTAPKPAFTALPSSSSSSSQKPFLRLALQFPSLPNSSYHSQRPSL-------KISSALSDA-------TAKTTTAAAAEDFVSRFGLEEPRKGADILVEALERQGV 95
BnALS1        ----MAAATSSSPISLTAK---------PSSKSPLPISRFSLPFSLTPQKDSSRLH-------RPLAISAVLNSPVNVA---PPSPEKTDKNKTFVSRYAPDEPRKGADILVEALERQGV 97
AtALS         MAAATTTTTTSSSISFSTK------PSPSSSKSPLPISRFSLPFSLNPNKSSSSSRRRGIKSSSPSSISAVLNTTTNVTTTPSPTKPTKPE--TFISRFAPDQPRKGADILVEALERQGV 112
CmALS1        --MAAATTTSSSSIPFSTK--------HSSSKSPLPISRFTLPFSLNPNKSSSSSSRRRGIKSTALSISAVLNTTTNVSTTTPQSKPTKPEKKKFVSRFAPDQPRKGADILVEALERQGV 110
CmALS2        --MAAATTTSSSSIPFSTK--------PSSSKSPLPISRFTLPFSLNPNKS---SSRRRGIKSTSLSISAVLNTTTNVSTTTPPSKPTKPEKKKFVSRFAPDQPRKGADILVEALERQGV 107
CrALS         ------------------------------------------------------------------------------------------------------------------------ 0
ClaALS        MAAAAS---PCASKSFSKP---------SSFSLPKPFSRFDLSFSPLPHNPSFRRPLRICSSSLSNPSPK--PSSTAASAAAVDTSTIA-SPEIFVSRFAADEPRKGADILVEALERQGV 105
Cs7g22130     MAAAS-----THTATFTNPPSSSFISTTSQKSALFPISKFTLPFSHKFGKPS----LRIITSSLPKPSAA--SSST------IAQTVITSPETNFSSRFAPDEPRKGADILVEALERQGV 103
EcALS         ------------------------------------------------------------------------------------------------------------------------ 0
EcALS3        -----------------------------------------------------------------------------------------------------MEMLSGAEMVVRSLIDQGV 19
BsALS         -----------------------------------------------------------------------MRTNVQ----------------VDSASAKCTQTMSGALMLIESLKKEKV 33
EcALS1        ----------------------------------------------------------------------------M----------------ASSGTSSTRKRFTGAEFIVHFLEQQGI 28
EcALS2        --------------------------------------------------------------------------------------------------------MNGAQWVVHALRAQGV 16

              ________________________________________________________________________________________________________________________
Cs5g35310     TTVFAYPGGASIEIHQSLTRSD-IRSILPRHEQGGIFAAEGYARSSGTPGVCLVSSGPGVTNIMTGLMDAYSDSIPILAITGQVSQKLLGTDAFQETPVVEVTRYMTKHNYLVLDVDDIP 204
PaALSa        SDVFAYPGGASMEIHQALTRSPAITNHLFRHEQGEAFAASGYARASGRVGVCVATSGPGATNLVSALADALLDSIPMVAITGQVPRRMIGTDAFQETPIVEVTRSITKHNYLVLDVEDIP 199
PaALSb        SDVFAYPGGASMEIHQALTRSPAITNHLFRHEQGEAFAASGYARASGRVGVCVATSGPGATNLVSALADALLDSIPMVAITGQVPRRMIGTDAFQETPIVEVTRSITKHNYLVLDVEDIP 204
OsALS2        RDVFAYPGGASMEIHQALTRSPVIANHLFRHEQGEAFAASGYARSSGRVGVCIATSGPGATNLVSALADALLDSVPIVAITGQVPRRMIGTDAFQETPIVEVTRSITKHNYLVLDVDDIP 200
SbALS1        RDVFAYPGGASMEIHQALTRSPVIANHLFRHEQGEAFAASGFARSSGRVGVCVATSGPGATNLVSALADALLDSVPMVAITGQVPRRMIGTDAFQETPIVEVTRSITKHNYLVLDVDDIP 203
GmALS2        TDVFAYPGGASMEIHQALTRSASIRNVLPRHEQGGVFAAEGYARSSGIPGVCIATSGPGATNLVSGLADAMLDSVPLVAITGQVPRRMIGTDAFQETPIVEVTRSVTKHNYLVLDVDDIP 215
BnALS1        ETVFAYPGGASMEIHQALTRSSTIRNVLPRHEQGGVFAAEGYARSSGKPGICIATSGPGATNLVSGLADAMLDSVPLVAITGQVPRRMIGTDAFQETPIVEVTRSITKHNYLVMDVDDIP 217
AtALS         ETVFAYPGGASMEIHQALTRSSSIRNVLPRHEQGGVFAAEGYARSSGKPGICIATSGPGATNLVSGLADALLDSVPLVAITGQVPRRMIGTDAFQETPIVEVTRSITKHNYLVMDVEDIP 232
CmALS1        ETAFAYPGGASMEIHQALTRSSSIRNVLPRHEQGGVFAAEGYARSTGKPGICIATSGPGATNLVSGLADALLDSVPLVAITGQVPRRMIGTDAFQETPIVEVTRSITKHNYLVMDVEDIP 230
CmALS2        ETVFAYPGGASMEIHQALTRSSSIRNVLPRHEQGGVFAAEGYARSTGKPGICIATSGPGATNLVSGLADALLDSVPLVAITGQVPRRMIGTDAFQETPIVEVTRSITKHNYLVMDVEDIP 227
CrALS         -----------MEIHQALTRSSSIRNVLPRHEQGGVFAAEGYARSSGKPGICIATSGPGATNLVSGLADALLDSVPLVAITGQVPRRMIGTDAFQETPIVEVTRSITKHNYLVMDVEDIP 109
ClaALS        TNVFAYPGGASMEIHQALTRSSIIRNVLPRHEQGGVFAAEGYARSSGLPGVCIATSGPGATNLVSGLADALLDSVPLVAITGQVPRRMIGTDAFQETPIVEVTRSITKHNYLVLDVDDIP 225
Cs7g22130     TDVFAYPGGASMEIHQALTRSNIIRNVLPRHEQGGIFAAEGYARSSGKPGICIATSGPGATNLVSGLADALLDSVPLVAITGQVPRRMIGTDAFQETPIVEVTRSITKHNYLVLDVDDIP 223
EcALS         -----------MEIHQALTRSTTIRNVLPRHEQGGIFAAEGYARASGLPGVCIATSGPGATNLVSGLADALLDSVPVVAITGQVPRRMIGTDAFQETPIVEVTRSITKHNYLVLDVDDIP 109
EcALS3        KQVFGYPGGAVLDIYDALHTVGGIDHVLVRHEQAAVHMADGLARATGEVGVVLVTSGPGATNAITGIATAYMDSIPLVVLSGQVATSLIGYDAFQECDMVGISRPVVKHSFLVKQTEDIP 139
BsALS         EMIFGYPGGAVLPIYDKLYNS-GLVHILPRHEQGAIHAAEGYARVSGKPGVVIATSGPGATNLVTGLADAMIDSLPLVVFTGQVATSVIGSDAFQEADILGITMPVTKHSYQVRQPEDLP 152
EcALS1        KIVTGIPGGSILPVYDALSQSTQIRHILARHEQGAGFIAQGMARTDGKPAVCMACSGPGATNLVTAIADARLDSIPLICITGQVPASMIGTDAFQEVDTYGISIPITKHNYLVRHIEELP 148
EcALS2        NTVFGYPGGAIMPVYDALYDG-GVEHLLCRHEQGAAMAAIGYARATGKTGVCIATSGPGATNLITGLADALLDSIPVVAITGQVSAPFIGTDAFQEVDILGLSLACTKHSFLVQSLEELP 135
                                                                                                            IPR012001
              __________________________________                                    ________________________________________________
Cs5g35310     RIIKEAFFLATSGRPGPVLIDIPVDVQLELA--VPNWNQPCKLPSCISSLPKEPDELALRQTLKLIVESKNPVLCVGGGCLNS--SEELRKFVGLTGIPVTCTTMGLGLFPCT-DELCLR 319
PaALSa        RVIQEAFFLASSGRPGPVLVDIPKDIQQQMA--VPVWDAPMSLPGYIARLPKPPATELLEQVLRLVGEARRPILYVGGGCSAS--GEELRRFVELTGIPVTTTLMGLGNFPSD-DPLSLR 314
PaALSb        RVIQEAFFLASSGRPGPVLVDIPKDIQQQMA--VPVWDAPMSLPGYIARLPKPPATELLEQVLRLVGESRRPILYVGGGSSAS--GEELRRFVELTGIPVTTTLMGLGNFPSD-DPLSLR 319
OsALS2        RVVQEAFFLASSGRPGPVLVDIPKDIQQQMA--VPAWDTPMSLPGYIARLPKPPATEFLEQVLRLVGESRRPVLYVGGGCAAS--GEELCRFVELTGIPVTTTLMGLGNFPSD-DPLSLR 315
SbALS1        RVVQEAFFLASSGRPGPVLVDIPKDIQQQMA--VPVWDTPMSLPGYIARLPKPPATELLEQVLRLVGESRRPVLYVGGGCAAS--GEELRRFVEMTGIPVTTTLMGLGNFPGD-DPLSLR 318
GmALS2        RIVNEAFFLATSGRPGPVLIDIPKDIQQQFA--IPNWDQPIRLPGYMSRLPKSPNENHLELIVRLVMESKKPVLYVGGGCLNS--SEELRRFVELTGVPVASTLMGLGAYPIA-DENSLQ 330
BnALS1        RIVQEAFFLATSGRPGPVLVDVPKDIQQQLA--IPNWDQPMRLPGYMSRLPQPPEVSQLGQIVRLISESKRPVLYVGGGSLNS--SEELGRFVELTGIPVASTLMGLGSYPCN-DELSLQ 332
AtALS         RIIEEAFFLATSGRPGPVLVDVPKDIQQQLA--IPNWEQAMRLPGYMSRMPKPPEDSHLEQIVRLISESKKPVLYVGGGCLNS--SDELGRFVELTGIPVASTLMGLGSYPCD-DELSLH 347
CmALS1        RIVEEAFFLATSGRPGPVLVDVPKDIQQQLA--IPNWEQSMRLPGYMSRMPKPPEDSHLEQIVRLISESKKPVLYVGGGCLNS--SEELGRFVELTGIPVASTLMGLGAYPCD-DELSLH 345
CmALS2        RIVEEAFFLATSGRPGPVLVDVPKDIQQQLA--IPNWEQSMRLPGYMSRMPKPPEDSHLEQIVRLISESKKPVLYVGGGCLNS--SEELGRFVELTGIPVASTLMGLGAYPCD-DELSLH 342
CrALS         RIVQEAFFLATSGRPGPVLIDIPKDIQQQLA--IPNWEQAMRLPGYMSRMPKPPEESHLEQIVRLISEAKKPVLYVGGGCLNS--SDELVRFVELTGIPVASTLMGLGSYPCD-AELSLH 224
ClaALS        RIVSEAFFLAISGRPGPVLIDIPKDVQQQLA--VPNWNQPMKLPGYLSRLPKPPIDSHLEQIVRLVSESKKPVLYVGGGCLNS--SEELRRFVKLTGIPVASTLMGLGAYPCS-GELSLQ 340
```

```
Cs7g22130  RIVREAFLLATSGRPGPVLIDVPKDIQQQLA--VPNWNQPIKLPGYMSRLPKEPSEVHLEQIVRLISESKKPVLYVGGGCLNS--SEELRKFVELTGIPVASTLMGLGSFPGT-DELSLQ 338
EcALS      RIVREAFYLARSGRPGPVLIDIPKDIQQQLC--VPKWDEPMRLPGYLSRLPKPPNDGLLEQIVRLVGESKRPVLYVGGGCLNS--SDELRRFVELTGIPVASTLMGLGSYPAS-SDLSLQ 224
EcALS3     QVLKKAFWLAASGRPGPVVVDLPKDILNPAKKLPYAWPEAVSMRSYNPTT--TGHKGQIKRALQTLVAAKKPVVYVGGGAITAGCHQQLKDTVEALNLPVVSSLMGLGAFPAT-HRQALG 256
BsALS      RIIKEAFHIATTGRPGPVLIDIPKDVATIEGEFS--YDHEMNLPGYQPTT--EPNYLQIRKLVEAVSSAKKPVILAGAGVLHGKASEELKNYVEQQQIPVAHTLLGLGGFPAD-HPLFLG 268
EcALS1     QVMSDAFRIAQSGRPGPVWIDIPKDVQTAVFEIEEQ----PGMTEKAAAP--AFSEESIRDAAAMINAAKRPVLYLGGGVINA--PARVRELAEKAQLPTTMTLMALGILPKA-HPLSLG 259
EcALS2     RIMAEAFDVASSGRPGPVLVDIPKDIQLASGDLEP------WFTTVENEV--TFPHAEVEQARQMLAKAQKPMLYVGGGVGMAQAVSALREFLAATKMPSTCTLKGLGAVDAD-YPYYLG 246

Cs5g35310  MVGMFGTVYANYAVNECDLLLAAGVRFNERMTSKLEDFATRAKIVHIDIDSNEIGKVKLPDVSICADAKLVFNRMNMIL-E----SKGVGFMFDFSAWREELHEQKKKYPFSY-KTFGEE 433
PaALSa     MLGMHGTVYANYAVDKADLLLAFGVRFDDRVTGKIEAFASRSKIVHIDIDPAEIGKNKQPHVSICADVKIALEGLNSLLLN----GSKTHKSLDFSSWHEELDQQKREFPLGF-KTFGEA 429
PaALSb     MLGMHGTVYANYAVDKADLLLAFGVRFDDRVTGKIEAFASRSKIVHIDIDPAEIGKNKQPHVSICADVKIALEGLNSLLLN----GSKTHKSLDFSSWHEELDQQKREFPLGF-KTFGEA 434
OsALS2     MLGMHGTVYANYAVDKADLLLAFGVRFDDRVTGKIEAFAGRAKIVHIDIDPAEIGKNKQPHVSICADVKLALQGMNTL-LE----GSTSKKSFDFGSWHDELDQQKREFPLGY-KIFNEE 429
SbALS1     MLGMHGTVYANYAVDKADLLLAFGVRFDDRVTGKIEAFASRAKIVHIDIDPAEIGKNKQPHVSICADVKLALQGMNAL-LE----GSTSKKSFDFGSWQAELDQQKREFPLGY-KTFDDE 432
GmALS2     MLGMHGTVYANYAVDKADILLAFGVRFDDRVTGKLEAFASRAKIVHIDIDSAEIGKNKQPHVSVCADLKLALKGINHML-E----SRGVGGKLDFRGWREELNEQKRRFPLSY-KTFEDE 444
BnALS1     MLGMHGTVYANYAVEHSDLLLAFGVRFDDRVTGKLEAFASRAKIVHIDIDSAEIGKNKTPHVSVCGDVKLALQGMNKVL-E----NRAEELKLDFGVWRSELSEQKQKFPLSF-KTFGEA 446
AtALS      MLGMHGTVYANYAVEHSDLLLAFGVRFDDRVTGKLEAFASRAKIVHIDIDSAEIGKNKTPHVSVCGDVKLALQGMNKVL-E----NRAEELKLDFGVWRNELNVQKQKFPLSF-KTFGEA 461
CmALS1     MLGMHGTVYANYSVEHSDLLLAFGVRFDDRVTGKLEAFASRAKIVHIDIDSAEIGKNKTPHVSVCGDVKLALQGMNKVL-E----NRAEELKLDFGVWRSELNEQKQKFPLSF-KTFGEA 459
CmALS2     MLGMHGTVYANYSVEHSDLLLAFGVRFDDRVTGKLEAFASRAKIVHIDIDSAEIGKNKTPHVSVCGDVKLALQGMNKVL-E----NRGEELKLDFGVWRSELNEQKQKFPLSF-KTFGEA 456
CrALS      MLGMHGTVYANYSVEHSDLLLAFGVRFDDRVTGKLEAFASRAKIVHIDIDSAEIGKNKTPHVSVCGDVKLALQGMNKVL-E----NRAEELKLDFGVWRNELNGQKQKFPLSF-KTFGEA 338
ClaALS     MLGMHGTVYANYAVDKSDLLLAFGVRFDDRVTGKLEAFASRAKIVHIDIDSAEIGKNKQPHVSVCGDVKLALQGMNRLL-E----K-TEKLHFDFSAWREELNEQKSKYPLTF-KTFDEA 453
Cs7g22130  MLGMHGTVYANYAVDKSDLLLAFGVRFDDRVTGKLEAFASRAKIVHIDIDSAEIGKNKQPHVSVCADVKLALKGINRIL-G----GKDAKGRFDFSAWREELDEQKVKYPLSF-KTFEEA 452
EcALS      MLGMHGTVYANYAVDKADLLLAFGVRFDERVTGKLEAFASRAKIVHIDIDSAEIGKNKQPHVSVCGDIKIALQGLNKIL-E----GRREMSNLDFSAWRAELDEQKVNHPLSF-KTFGEA 338
EcALS3     MLGMHGTYEANMTVHNADVIFAVGVRFDDRTTNNLAKYCPNATVLHIDIDPTSISKTVAADIPIVGDARQVLEQMLELLSQ----ESAHQPLDEIRDWWQQIEQWRARQCLKY-DTHSEK 371
BsALS      MAGMHGTYTANMALHECDLLISIGARFDDRVTGNLKHFARNAKIAHIDIDPAEIGKIMKTQIPVVGDSKIVLQELIKQDGK----------QSDSSEWKKQLAEWKEEYPLWYVDNEEEG 377
EcALS1     MLGMHGVRSTNYILQEADLLIVLGARFDDRAIGKTEQFCPNAKIIHVDIDRAELGKIKQPHVAIQADVDDVLAQLIPQVEA-----------QPRAEWHQLVADLQREFPCPI-PKACDP 367
EcALS2     MLGMHGTKAANFAVQECDLLIAVGARFDDRVTGKLNTFAPHASVIHMDIDPAEMNKLRQAHVALQGDLNALLPALRQPL-------------NINDWQQHCAQLRDEHAWRY-NHPGDA 351
                                                                                  IPR000399
                              IPR011766
Cs5g35310  IPPQYAIQILNELTDDEETIISTGVGQHQMWAIQFYMYKRARQLLTSSGFGSMGFGLPAAMGAAVANPGAIVVDIDGDGSFIMNLQELAAIKAENIPVKILLINNQYLGMNVEYEDRYFE 553
PaALSa     IPPQYAIQVLDELTKG-EAIIATGVGQHQMWAAQYYTYKRPRQWLSSAGLGAMGFGLPAAAGAAVANPGVTVVDIDGDGSFLMNIQELALIRIENLPVKVMILNNQHLGMVVQWEDRFYK 548
PaALSb     IPPQYAIQVLDELTKG-EAIIATGVGQHQMWAAQYYTYKRPRQWLSSAGLGAMGFGLPAAAGAAVANPGVTVVDIDGDGSFLMNIQELALIRIENLPVKVMILNNQHLGMVVQWEDRFYK 553
OsALS2     IQPQYAIQVLDELTKG-KAIIATGVGQHQMWAAQYYTYKRPRQWLSSAGLGAMGFGLPAAAGAAVANPGVTVVDIDGDGSFLMNIQELAMIRIENLPVKVFVLNNQHLGMVVQWEDRFYK 548
SbALS1     IQPQYAIQVLDELTKG-EAIIATGVGQHQMWAAQYYTYKRPRQWLSSAGLGAMGFGLPAAAGAAVANPGITVVDIDGDGSFLMNIQELAMIRIENLPVKVFVLNNQHLGMVVQWEDRFYK 551
GmALS2     ISPQYAIQVLDELTNG-DAIVSTGVGQHQMWAAQFYKYKRPRQWLTSGGLGAMGFGLPAAIGAAVANPGAVVVDIDGDGSFIMNVQELATIKVEKLPVKILLLNNQHLGMVVQWEDRFYK 563
BnALS1     IPPQYAIQILDELTEG-KAIISTGVGQHQMWAAQFYKYRKPRQWLSSSGLGAMGFGLPAAIGASVANPDAIVVDIDGDGSFIMNVQELATIRVENLPVKILLLNNQHLGMVMQWEDRFYK 565
AtALS      IPPQYAIKVLDELTDG-KAIISTGVGQHQMWAAQFYNYKKPRQWLSSGGLGAMGFGLPAAIGASVANPDAIVVDIDGDGSFIMNVQELATIRVENLPVKVLLLNNQHLGMVMQWEDRFYK 580
CmALS1     IPPQYAIQVLDELTDG-RAIISTGVGQHQMWAAQFYKYKKPRQWLSSAGLGAMGFGLPAAIGASVANPDSIVVDIDGDGSFIMNVQELATIRVENLPVKILILNNQHLGMVMQLEDRFYK 578
CmALS2     IPPQYAIQVLDELTDG-KAIISTGVGQHQMWAAQFYKYKKPRQWLSSAGLGAMGFGLPAAIGASVANPDAVVVDIDGDGSFIMNVQELATIRVENLPVKILILNNQHLGMVMQWEDRFYK 575
CrALS      IPPQYAIQVLDELTDG-KAIISTGVGQHQMWAAQFYKYKKPRQWLTSAGLGAMGFGLPAAIGASVANPHAIVIDIDGDGSFMMNLQELATIRVENLPVKILLLNNQHLGMVMQWEDRFYK 457
ClaALS     IPPQYAIQLLDELTNG-EAIVSTGVGQHQMWAAQFYKYKKPRQWLTSGGLGAMGFGLPAAMGAAVANPDAVVVDIDGDGSFIMNVQELATISVEKLPVKILLLNNQHLGMVVQWEDRFYK 572
Cs7g22130  IPPQYAIQVLDELTNG-EAIISTGVGQHQMWAAQFYKYRRPRQWLTSGGLGAMGFGLPAAIGAAVANPDAIIVDIDGDGSFIMNVQELATIKVEKLPIKILLLNNQHLGMVMQWEDRFYK 571
EcALS      IPPQYAIXVLDELTGG-NAIISTGVGQHQMWAAQFYKYNRPRQWLTSGGLGAMGFGLPAALGVKMALPEETVVCVTGDGSIQMNIQELSTALQYELPVLVVNLNNRYLGMVKQWQDMIYS 490
EcALS3     IKPQAVIETLWRLTKG-DAYVTSDVGQHQMFAALYYPFDKPRRWINSGGLGTMGFGLPAALGVKMALPEETVVCVTGDGSIQMNIQELSTALQYELPVLVVNLNNRYLGMVKQWQDMIYS 490
BsALS      FKPQKLIEYIHQFTKG-EAIVATDVGQHQMWSAQFYPFQKADKWVTSGGLGTMGFGLPAAIGAQLAEKDATVVAVVGDGGFQMTLQELDVIRELNLPVKVVILNNACLGMVRQWQEIFYE 496
EcALS1     LSHYGLINAVAACVDD-NAIITTDVGQHQMWTAQAYPLNRPRQWLTSGGLGTMGFGLPAAIGAALANPDRKVLCFSGDGSLMMNIQEMATASENQLDVKIILMNNEALGLVHQQQSLFYK 486
EcALS2     IYAPLLLKQLSDRKPE-DCVVTTDVGQHQMWAAQHIVHTRPENFITSSGLGTMGFGLPAAVGAQVARPNDTVVCISGDGSFMMNVQELGTVKRKQLPLKIVLLDNQRLGMVRQWQQLFFQ 470

Cs5g35310  ANRANSFLGDPLRKSEIFPDMLKFAEACGIPAARVTKKKDVRAAIQLMLE---TPGPYLLDVMVSYQEHVVPMIPYDKSFKDTILEDDGRALH-----      643
PaALSa     ANRAHTYLGNPENESEIYPDFVTIAKGFNVPAVRVTKKSEVRAAIKKMLE---TPGPYLLDIIVPHQEHVLPMIPSGGAFKDIIMDGDGRIAY-----      638
PaALSb     ANRAHTYLGNPENESEIYPDFVTIAKGFNVPAVRVTKKSEVRAAIKTMLE---TPGPYLLDIIVPHQEHVLPMIPSGGAFKDIIMEGDGRIA------      642
OsALS2     ANRAHTFLGNPENESEIYPDFVAIAKGFNIPAVRVTKKSEVHAAIKKMLE---APGQYLLDIIVPHQEHVLPMIPSGGAFKDMILDGDGRTVY-----      638
```

```
SbALS1      ANRAHTYLGNPENESEIYPDFVTIAKGFNIPAVRVTKKSEVHAAIKKMLE---TPGPYLLDIIVPHQEHVLPMIPSGGAFKDMILDGDGRTVY-----    641
GmALS2      SNRAHTYLGDPSNENAIFPDMLKFADACGIPAARVTKKEDLRAAIQKMLD---TPGPYLLDVIVPHQEHVLPMIPSNGTFQDVITEGDGRTSY-----    653
BnALS1      ANRAHTYLGDPARENEIFPNMLQFAGACGIPAARVTKKEELREAIQTMLD---TPGPYLLDVICPHQEHVLPMIPSGGTFKDVITEGDGRTKY-----    655
AtALS       ANRAHTFLGDPAQEDEIFPNMLLFAAACGIPAARVTKKADLREAIQTMLD---TPGPYLLDVICPHQEHVLPMIPSGGTFNDVITEGDGRIK------    669
CmALS1      ANRAHTYLGNPAEEDEIFPNMLQFASACGIPAARVTKIAELREAIQKMLD---TPGPYLLDVICPHQEHVLPMIPSGGTFNDVITEGDGRTKY-----    668
CmALS2      ANRAHTYLGNPATEDEIFPNMLQFASACGIPSARVTKKAELREAIQKMLD---TPGPYLLDVICPHQEHVLPMIPSGGTFNDVITEGDGRTKYEMKLT    670
CrALS       ANRAHTYLGNPAEEDKIFPNMLEFASACGIPAARVTKKEQLREAIQKMLD---TPGPYLLDVICPHQEHVLPMIPSGGTFNDVITEGDGRTK------    546
ClaALS      ANRAHTYLGDPSNETEIFPNMLKFAEACGIPAARVTKRAELRAAMKKMLE---TEGPYLLDVIVPHQEHVLPMIPSGGAFKDVITEGDGRSVY-----    662
Cs7g22130   ANRAHTYLGDPSRESEIFPNMLKFAEACGIPGAQVTRKADLRAAIQTMLD---TPGPYLLDVIVPHQEHVLPMIPSGGAFKDVITEGDGRTQYRQ---    663
EcALS       ANRAHTYLGNPSKEAEIFPNMLKFAEACDIPAARVTRKADLRAAIQKMLD---TPGPYLLDVIVPHQEHVLPMIPAGGGFMDVITEG-----------    541
EcALS3      GRHSQSYMQS-------LPDFVRLAEAYGHVGIQISHPQELESKLSEALEQVRNNRLVFVDVTVDGSEHVYPMQIRGGGMDEMWLSKTERT-------    574
BsALS       ERYSESKFAS-------QPDFVKLSEAYGIKGIRISSEAEAKEKLEEALT---SREPVVIDVRVASEEKVFPMVAPGKGLHEMVGVKP----------    574
EcALS1      QGVFAATYPG-------KINFMQIAAGFGLETCDLNNEADPQTALQEIIN---RPGPALIHVRIDAEEKVYPMVPPGAANTEMVGE------------    562
EcALS2      ERYSETTLTD-------NPDFLMLASAFGIPGQHITRKDQVEAALDTMLN---SDGPYLLHVSIDELENVWPLVPPGASN------------------    540
```
