# Supplementary material for: Generation of Transfer-DNA-Free Base-Edited Citrus Plants
Source: Front Plant Sci. 2022 Mar 15;13:835282. doi: 10.3389/fpls.2022.835282 (PMC8965368; doi:10.3389/fpls.2022.835282)
Supplement: Supplementary file 5 [file Data_Sheet_1.docx]

**Table S1**. Base editing activity against gALS target sequence

| nº | |  |  |
| --- | --- | --- | --- |
| seq | mut | seq (5’ 🡪 3’) |  |
| 10 | 0 | GCCTATGATCCCCAGTGG |  |
| 4 | 1 | GCCTATAATCCCCAGTGG |  |
| 1 | 1 | GCCTATGATCCCCAATGG |  |
| 1 | 1 | GCCTATGATCCCCAGTGA |  |
| 1 | 2 | GCCTATAATCTCCAGTGG |  |
| 2 | 2 | ATCTATGATCCCCAGTGG |  |
| 5 | 2 | GTCTATAATCCCCAGTGG |  |
| 1 | 2 | ACCTATGATCCCCAGTGA |  |
| 2 | 3 | GTCTATAATCTCCAGTGG |  |
| 1 | 3 | GTCTATAATTCCCAGTGG |  |
| 1 | 3 | GTCTATAATCCTCAGTGG |  |
| 1 | 3 | ATCTATAATCCCCAGTGG |  |
| 1 | 3 | ATCTATGATCTCCAGTGG |  |
| 1 | 3 | GCCTATAATTTCCAGTGG |  |
| 1 | 4 | GTCTATAATTTCCAGTGG |  |
| 1 | 4 | GTCTATAATCTTCAGTGG |  |
| 1 | 4 | ACCTATAATCCCTAGTAG |  |
| 1 | 4 | ACCTATAATCTCCAATGG |  |
| 1 | 4 | GCCTATAATCTCTAATGG |  |
| 1 | 5 | GTCTATGATCTCTAATGA |  |
| 1 | 6 | GTCTATGATCTTTAATGA |  |
| 1 | 7 | ACTTATAATTCTTAGTGA |  |
| 1 | 7 | ACTTATAATTTTCAATGG |  |

**Table S2.** Primers used in this study. Nucleotides used to incorporate sequences of interest into Golden Braid system are red-lettered, while those directed to introduce substitutions in ALSm clones are low-lettered.

| **Name** | **Sequence (5´🡪 3´)** |
| --- | --- |
| B226 | GCGCCGTCTCGCTCGAATGGCGGCCGCTTCGACGC |
| B227 | GCGCCGTCTCGCTCGAAGCTTAATACTGCGTTCTTCCATCA |
| B228 | GCACGGACGtTTTTCAAGAG |
| B229 | CTCTTGAAAAaCGTCCGTGC |
| B230 | CAAGTACAGGcGACCTCGCC |
| B231 | GGCGAGGTCgCCTGTACTTG |
| B232 | GTGATGCAATtGGAGGATCG |
| B233 | CGATCCTCCaATTGCATCAC |
| B234 | TGATCCCCAaTGGTGGAG |
| B235 | CTCCACCAtTGGGGATCA |
| pJET-F | CGACTCACTATAGGGAGAGCGGC |
| pJET-R | AAGAACATCGATTTTCCATGGCAG |
| B413 | GTGCACCACTGGGGATCATAGGC |
| B414 | AAACGCCTATGATCCCCAGTGGt |
| B13R | AATCGAGCTCGGCAATTCCCGATCT |
| 35S finalF | CACAATCCCACTATCCTTCG |
| GUSup | GGTGGGAAAGCGCGTTACAA |
| GUSdown | TGGATTCCGGCATAGTTAAA |
| B599 | CCTGAACTAAAAACACTGCC |
| B596 | TCTCGTGCTTCTTGTCCTCC |
| B595 | AGTCTAAGAACGGCTACG |
| B600 | ATAGTCTCCTCTGACTTCC |
| B421 | ATGCGAATCTCGACAAGG |
| B422 | TGACGAACGTTGTCGAAACC |
| MU20 | GCGCGGTCTCGGGAGCCAATAAGCTTGCATGCC |
| MU21 | GCGCGGTCTCGAGCGTTCGTTGTCAATCAATTGGCAAG |
|  |  |

**Table S3.** Predicted on-target analysis from Benchling using *C. sinensis* genome (GenBank GCA_000317415.1) as reference.

| **Position** | **Guide strand** | **Sequence** | **# edits** | **Base-editing scores** | **Off-target score** |
| --- | --- | --- | --- | --- | --- |
| 19616063 | + | TATTATTGGGGATCATAGGC | 4 | 0.5, 5.6, 9.3, 13.5 | 97.7 |
|  |  |  |  |  |  |

**Table S4.** Off-targets scores derived from Benchling analysis using *C. sinensis* genome (GenBank GCA_000317415.1) as reference. Base mismatches are denoted in red.

| **Sequence** | **PAM** | **Score** | **Locus** |
| --- | --- | --- | --- |
| CACCACTGGGGATCATAGGC | AGG | 100 | Chr7:+19616080 |
| CCCCACTAGGGTTCATAGGC | TGG | 1.5 | Scaffold_0123:-544151 |
| CAACACTGTTGATCAAAGGC | AAG | 0.2 | Chr1:-235231 |
| CAACACTGTTGATCAAAGGC | AAG | 0.2 | Chr1:-233379 |
| CAGCAATGAGGATCATAAGC | TGG | 0.1 | Scaffold_0132:+130010 |
| CAGCAATGAGGATCATAAGC | TGG | 0.1 | Chr7:+19157688 |
| GACCACTAGGGTTTATAGGC | CGG | 0.1 | Scaffold_0093:+165027 |
| CACCACAGTGCATCATAAGC | AGG | 0.1 | Chr6:+9171880 |
| CACCACTGGGCAGCTAAGGC | CGG | 0.0 | Scaffold_0206:-284789 |
|  |  |  |  |
